# The application of remote ischemic conditioning in cardiac surgery

**DOI:** 10.12688/f1000research.11018.1

**Published:** 2017-06-16

**Authors:** Zeljko J. Bosnjak, Zhi-Dong Ge

**Affiliations:** 1 Department of Anesthesiology, Medical College of Wisconsin, 8701 Watertown Plank Road, Milwaukee, WI, 53226, USA; 2 Department of Physiology, Medical College of Wisconsin, 8701 Watertown Plank Road, Milwaukee, WI, 53226, USA

**Keywords:** ischemia, reperfusion, cardioprotective agents

## Abstract

Perioperative myocardial ischemia and infarction are the leading causes of morbidity and mortality following anesthesia and surgery. The discovery of endogenous cardioprotective mechanisms has led to testing of new methods to protect the human heart. These approaches have included ischemic pre-conditioning, per-conditioning, post-conditioning, and remote conditioning of the myocardium. Pre-conditioning and per-conditioning include brief and repetitive periods of sub-lethal ischemia before and during prolonged ischemia, respectively; and post-conditioning is applied at the onset of reperfusion. Remote ischemic conditioning involves transient, repetitive, non-lethal ischemia and reperfusion in one organ or tissue (remote from the heart) that renders myocardium more resistant to lethal ischemia/reperfusion injury. In healthy, young hearts, many conditioning maneuvers can significantly increase the resistance of the heart against ischemia/reperfusion injury. The large multicenter clinical trials with ischemic remote conditioning have not been proven successful in cardiac surgery thus far. The lack of clinical success is due to underlying risk factors that interfere with remote ischemic conditioning and the use of cardioprotective agents that have activated the endogenous cardioprotective mechanisms prior to remote ischemic conditioning. Future preclinical research using remote ischemic conditioning will need to be conducted using comorbid models.

## Introduction

When the coronary arterial blood flow is blocked, it is critical to re-establish the blood flow to the ischemic area of the heart as soon as possible. During the return of blood supply to the ischemic myocardium, there is paradoxical myocardial damage; the excess oxygen may trigger further myocardial cell death and greater cardiac injury, termed myocardial reperfusion injury ^1^. Although medical advances in cardiac treatment have been significant, there is very little that can be done pharmacologically or mechanically to prevent the injury of the reperfused myocardium, which can lead to heart failure and death. The development of novel cardioprotective strategies that would mitigate further myocardial injury, secondary to ischemia/reperfusion (I/R) injury, is required and is the focus of many preclinical and clinical investigations ^2, 3^. The endogenous cardioprotection—mediated via ischemic pre-conditioning, ischemic per-conditioning, and ischemic post-conditioning—has some clear drawbacks because it has to be administered to the compromised myocardium. On the other hand, the maneuvers implemented with remote ischemic conditioning (RIC) (pre-, per-, and post-conditioning) have the advantage of applying the protective ischemia to an organ distant from the heart ^4^. Therefore, in this short review article, we focus mostly on RIC because of its popularity, non-invasive nature, and relative safety.

## Potential mechanisms underlying cardioprotection by remote ischemic conditioning

RIC is the phenomenon in which transient, repetitive, non-lethal ischemia and reperfusion in one organ or tissue (remote from the heart) render myocardium resistant to lethal I/R injury ^4^. It represents a strategy for harnessing the body’s endogenous, protective capabilities against the myocardial injury incurred by I/R. In experimental animals, brief episodes of ischemia and reperfusion in an arm or a leg dramatically reduce myocardial infarct size when applied prior to lethal myocardial ischemia (remote ischemic pre-conditioning), during lethal myocardial ischemia (remote ischemic per-conditioning), or at the onset of reperfusion (remote ischemic post-conditioning) ^5– 10^. Remote ischemic pre-conditioning and post-conditioning produce similar efficacy of cardioprotection against I/R injury ^11^.

The mechanisms of cardioprotection by RIC are complex and have not been fully elucidated. However, it has been established that the signaling pathways from the tissue/organ subject to transient, repetitive I/R to the heart consist of three entities: remote stimulus to generate protective signal, the transfer of the signal to the heart, and myocardial responses to the transferred signal resulting in cardioprotection ^12, 13^. In experimental animals, if the sensory nerve to the ischemic limb, spinal cord, or the vagus nerve is transected or silenced, the cardioprotective effects of RIC are lost ^14– 17^. These studies reveal the importance of neural pathways in transmitting the signal for cardioprotection in RIC. Over the past few years, many humoral factors have also been implicated, including nitric oxide, adenosine, bradykinin, opioid peptides, prostaglandins, natriuretic peptides, endocannabinoids, angiotensin I and calcitonin gene-related peptide, hypoxia-inducible factor 1α, erythropoietin, stromal-derived factor 1α, hypoxia-inducible factor prolyl hydroxylase 2 (encoded by *EGLN1* gene), and microRNAs ^18– 24^. Currently, it is believed that the stimulus of remote ischemic pre-conditioning activates afferent C fiber sensory nerves by locally released autacoids to transmit cardioprotective signal ^25^. In the meantime, the aforementioned humoral factors released by the tissue undergoing transient I/R are relayed in the blood to the heart, where they trigger cardioprotection. Humoral pathways may be more prominent in remote ischemic post-conditioning relative to remote ischemic pre-conditioning ^11, 14^.

The intracellular signaling pathways of RIC in myocardium are thought to have much in common with local ischemic pre- and post-conditioning ^26^. Autacoids, neurohormones, and humoral factors generated by remote stimulus bind to G protein-coupled receptors in myocardium or activate intracellular signal pathways in a receptor-independent manner or do both ^25^. Three main intracellular signaling pathways may be critical in RIC-elicited cardioprotection against I/R injury: the endothelial nitric oxide synthase/protein kinase G pathway, the reperfusion injury salvage kinase pathway, and the survivor activating factor enhancement pathway ^13, 27^. Ultimately, these signaling pathways converge on the mitochondria and the cytoskeleton, resulting in inhibition of opening of the mitochondria permeability transition pore (mPTP), preservation of mitochondrial integrity and function, and reduction of cytoskeleton damage ^28, 29^.

It is worth noting that there is considerable similarity between remote conditioning and exercise. Exercise appears to act as a physiological stress leading to similar accumulation of metabolic mediators such as adenosine, bradykinin, and calcitonin gene-related peptide along with baroreflex responses that induce beneficial myocardial adaptive responses at a cellular level ^30^. Moreover, it was shown that individuals with cardiovascular disease who participate in vigorous exercise prior to their cardiac event have improved ejection fraction and may obtain a protective benefit similar to that of RIC ^31^.

## Clinical trials of remote ischemic conditioning for cardiac surgery

RIC was shown to reduce cardiac injury in patients undergoing revascularization and other cardiac surgeries, as seen by reduction of cardiac biomarker release ^32– 36^. Notwithstanding more recent clinical outcome studies (for example, ERICCA and RIPHeart), most of the clinical studies were conducted on small cohorts of selected patients and in controlled conditions. These smaller trials on the effectiveness of RIC during elective interventional revascularization, other forms of cardiac surgeries, non-cardiac vascular surgeries, and others have shown various degrees of beneficial effects of RIC ^37, 38^.

One of the larger trials, which involved 1,280 patients and used both remote ischemic pre- and post-conditioning, did not show a reduction in the number of major adverse outcomes ^39^. Some additional negative studies on RIC in humans were attributed mostly to underlying risk factors or medications that interfere with different cardioprotective interventions. One recent review covered the cardioprotection by ischemic pre-conditioning, ischemic post-conditioning, and remote conditioning in various clinical settings ^40^. In addition to comorbidities and medications, the negative results may be due to the anesthetics used during the surgeries, which have been proven to prevent the protection by RIC. For instance, propofol has been shown to eliminate the benefits of RIC. It is of interest that most of the negative studies on the protection of RIC so far have used propofol as anesthetic ^41^.

Owing to both positive and negative results from various clinical trials, the results from large multicenter randomized controlled trials (such as ERICCA and RIPHeart) were meant to close that gap ^42^. In the ERICCA study, with 1,612 patients undergoing elective on-pump coronary artery bypass grafting with or without valve surgery and without standardization of the anesthetic regimen, remote ischemic pre-conditioning using transient-arm I/R did not improve clinical outcomes ^43^. Similarly, the RIPHeart study, in which the upper-limb remote ischemic pre-conditioning was performed while 1,385 patients were under propofol-induced anesthesia, did not show a relevant benefit among patients undergoing elective cardiac surgery ^44^. The trials also failed to confirm the presence of initial cardioprotection by RIC-induced reduction of cardiac troponin release. Both of these studies are a clear disappointment in the cardioprotection efforts ^45^. These large trials (ERICCA and RIPHeart) during cardiac surgery contradict previous smaller trials on the role of RIC ^34, 36^. Taken together, the recent large multicenter trials using RIC have not proven successful in cardiac surgery.

The lack of success in cardiac surgery using RIC is multifactorial. One cause is that cardiovascular risk factors can interfere with RIC. Most of the risk factors responsible for human ischemic heart disease in the first place include hypertension, chronic obstructive pulmonary disease, heart failure, atherosclerosis, diabetes and other metabolic diseases, age, and routine drug therapies ^40, 46– 48^. Another cause is the utility of cardioprotective agents that have activated the endogenous cardioprotective mechanisms prior to RIC ^49^. Potential cardioprotective agents that would mitigate/interfere with cardioprotective interventions may include volatile anesthetics, propofol, P2Y12 blocking agents, beta blockers, morphine, nicorandil, sulfonylureas, statins, angiotensin-converting enzyme inhibitors, and nitrates ^49– 53^. In addition, the beneficial effect of ischemic conditioning is not detectable in patients with the small extent of myocardial injury ^54^.

ST segment elevation myocardial infarction is one of the leading causes of mortalities and morbidities worldwide ^55,56^, and infarct size is the main determinant of prognosis. Reduction of infarct size is a main goal of treatment and can be achieved efficiently with primary angioplasty. Successful and timely reperfusion with percutaneous coronary intervention (PCI) or primary angioplasty attenuates infarct size and improves cardiac function and clinical outcomes ^57, 58^. However, sudden reperfusion can cause fatal myocardial injury ^2, 3, 59^, which may limit therapeutic benefits. Thus, supplementary cardioprotection such as RIC may be considered in elective and emergent PCI ^60^. Clinical trials using small cohorts of selected patients generally suggest that RIC can provide cardioprotection by lowering peak troponin I or reducing infarct size (or both) in patients undergoing elective PCI ^60– 64^.

## Effects of anesthetic cardioprotection on remote ischemic conditioning in cardiac surgery

One could speculate that the lack of protection in these two phase III clinical trials is because of the use of propofol anesthesia in most of the patients in the ERICCA trial and all patients in the RIPHeart trial. Attenuation of RIC in the presence of propofol anesthesia has been reported ^65^, and the use of propofol, rather than volatile anesthesia, appears to be a common denominator of studies that failed to protect with RIC ^66, 67^. It is likely that the use of volatile anesthetics would have made the ERICCA and RIPHeart trials more complete. Indeed, the successful cardioprotection by RIC was also documented in acute myocardial infarction, where the type of anesthesia was not an issue ^60, 68, 69^.

Parallel to the most powerful endogenous cardioprotective mechanism of ischemic pre-conditioning ^70^, pharmacologic cardioprotection with volatile anesthetics emerged as a considerably less risk-bearing and equally effective intervention ^71^. The American College of Cardiology Foundation and the American Heart Association Task Force on Practice Guidelines adopted a recommendation for the use of volatile anesthetics in surgical patients at risk for myocardial ischemia ^72^. Since anesthetic cardioprotection was discovered, experimental and clinical research has focused on elucidating the mechanisms of anesthetic cardioprotection with the anticipation of finding an anesthetic agent or approach that would be the most beneficial for patients with coronary artery disease. Clinical studies with sufficient power to detect differences between process variables and outcome among anesthetic agents or techniques have confirmed the relevance of anesthetic cardioprotection for patients ^73– 79^. The loss of cardioprotection is strongly associated with the risk of death/non-fatal myocardial infarction within the year after determining the absence of pre-conditioning with the PCI model of coronary occlusion ^80^. At one year, the risks of death were reduced by 85% in patients who manifested pre-conditioning as compared with patients who did not. The ability for cardioprotection to remain significantly, and inversely, associated with the risk of death/non-fatal myocardial infarction in one year affirms the clinical significance of this phenomenon.

Every year, volatile anesthetics are used in millions of patients undergoing cardiac surgery. Whether RIC provides more cardioprotection to the myocardium of the volatile anesthetic-anesthetized patients undergoing cardiac surgery has been examined, yet the clinical outcomes remain uncertain. Some clinical studies show that the cardioprotective effect of RIC is unable to be detected in isoflurane-anesthetized patients undergoing coronary artery bypass grafting ^50, 51^. In contrast, other small-scale clinical trials indicate that RIC can provide additional protective effects in isoflurane-anesthetized patients undergoing cardiac surgery ^41, 67^. However, recent studies from two large-scale, multicenter, clinical trials of RIC show negative results in cardiac surgery ^43, 44^. The reasons for the differences among these studies are complex and have not been fully understood. To use better RIC and lessen myocardial I/R injury in human cardiac surgery, a greater understanding of the interaction between RIC and volatile anesthetic conditioning is necessary.

## Challenges of remote ischemic conditioning in diabetic cardiovascular disease

Diabetes is a significant predictor of increased perioperative risk due to a greater susceptibility to I/R injury. Both preclinical and clinical results indicate that the cardioprotective effect of ischemic and pharmacologic conditioning is impaired in the presence of diabetes. Type 2 diabetes occurs in 9.3% of the US population, affecting 29.1 million individuals ^81^; and the prevalence of this disease is expected to increase by over 200% in the next several decades. Impaired glucose tolerance currently affects 20% to 35% of all middle-aged and elderly Americans, and hyperglycemia alone is a significant independent predictor of cardiovascular morbidity and mortality in patients undergoing cardiac surgery ^82– 85^. The mechanisms that contribute to increased risk in diabetes and hyperglycemia are poorly understood but likely are related to insufficient activation of pro-survival signaling pathways, elevated nitrosative stress, activation of the PI3K/Akt/mTOR (phosphatidylinositol 3-kinase, serine/threonine kinase also known as protein kinase B, and mammalian target of rapamycin) pathway, and autophagy ^46^. Aggressive control of blood glucose concentration using insulin is one approach, but it is unlikely that insulin alone can substantially improve cardiovascular outcomes in patients with diabetes.

Patients with diabetes have a significantly higher incidence of coronary heart disease compared with non-diabetic individuals ^55, 86^. At present, 15% to 30% of the patients who undergo coronary artery surgery are diabetic ^87– 89^. During cardiac surgery, the heart is inevitably subjected to I/R injury due to pre-existing coronary occlusion and heart arrest subsequent to aortic artery cross-clamp. After cardiac surgery, patients with diabetes have an increased mortality and poorer clinical recovery than non-obese, non-diabetic patients ^55, 90^. Therefore, diabetic populations may obtain a greater benefit from therapies shown to be effective in treating ischemic heart disease.

The cardioprotective effect of single-dose RIC in diabetes is often disappointing ^46, 91– 93^. The mechanisms underlying the impaired cardioprotective effect of RIC by diabetes are not fully elucidated. About 60% to 70% of people with diabetes mellitus eventually develop diabetic peripheral neuropathy ^94^. In many of these patients, peripheral neuropathy, including sensory C fibers which are required by the cardioprotective effect of RIC, is damaged ^95^. This damage may be an important contributor to the reduction of RIC cardioprotection during diabetes ^96, 97^. In future clinical trials, selection of patients with functional sensory C fibers may help to improve the clinical outcome of RIC. In addition, diabetes impairs the PI3K/Akt/glycogen synthase kinase 3 beta (PI3K/Akt/GSK3-β) signaling pathway and phosphorylation of ERK1/2 (extracellular signal-regulated protein kinases 1 and 2), decreases generation and release of nitric oxide, inactivates ATP-sensitive potassium channels, and elevates oxidative stress ^98– 100^. These intracellular signaling pathways are crucial for endogenous cardioprotection. Diabetes-elicited impairments in these signaling pathways may critically contribute to the attenuation of RIC cardioprotection ^92^.

Repeated RIC, where short periods of limb ischemia are repeatedly applied over days or weeks, is the extension of RIC. Recent studies have identified repeated RIC as a newer strategy for cardioprotection ^27, 93, 101, 102^. Compared with clinical single-dose RIC and local ischemic conditioning, repeated RIC appeared to have more consistently yielded significantly beneficial results against remodeling in both preclinical and clinical studies of RIC ^101– 103^. It has been demonstrated that repeated RIC reduces adverse cardiac remodeling after myocardial infarction, elevates survival of animals in a dose-dependent fashion, improves endothelial function and skin microcirculation, and modulates the systemic inflammatory responses ^101, 104, 105^. Intriguingly, repeated RIC is beneficial for healing in lower-extremity diabetic ulcers ^106^. Whether repeated RIC is effective in I/R protection of diabetic hearts remains elusive. The efficacy, potential, and safety of repeated RIC in protection of diabetic hearts need to be tested in future experimental studies and clinical trials.

Restoring myocardial sensitivity to RIC in the setting of diabetes is of primary importance. Since diabetes impairs multiple signal transduction pathways of RIC, it is reasonable to believe that a therapy that targets one pathway may not completely restore the sensitivity of the myocardium to RIC. In the various signal transduction pathways, GSK3-β is the intervention point of convergence, and the mPTP is thought to be the final effector of cardioprotection ^107, 108^. The studies to restore myocardial sensitivity to local ischemic conditioning in diabetes have focused on GSK3-β and the mPTP. Pharmacological inhibition of either GSK3-β or the mPTP restores the protective potential of local ischemic conditioning in the diabetic heart ^109, 110^. Whether pharmacological interventions also are effective in restoring the cardioprotective potential of RIC in diabetes has not been investigated. Given that intact neural pathway is required for the cardioprotective effect of RIC ^13^, it is likely that a combination of approaches that target both neural integrity and the final common signaling molecules and effectors would be the best strategy for restoring the myocardial response to RIC in diabetes.

## Summary

It is fair to say that owing to underlying risk factors that interfere with different cardioprotective interventions and the use of cardioprotective agents, most of the clinical trials with cardioprotective drugs have not been very successful. The results of various ischemic conditioning in humans appear to follow the same unsuccessful path, although RIC is a potent form of endogenous cardioprotection in healthy animals. In future research endeavors, the validation of drug targets and various cardiac conditioning needs to be conducted in comorbid animal models to have a successful clinical translation.

## Abbreviations

ERICCA, Effect of Remote Ischemic Preconditioning on Clinical Outcomes in Patients Undergoing Coronary Artery Bypass Surgery; GSK3-β, glycogen synthase kinase 3 beta; I/R, ischemia/reperfusion; mPTP, mitochondria permeability transition pore; PCI, percutaneous coronary intervention; PI3K/Akt, phosphatidylinositol 3-kinase, serine/threonine kinase also known as protein kinase B; RIC, remote ischemic conditioning; RIPHeart, Remote Ischemic Preconditioning for Heart Surgery.
